# Genetic and Phenotypic Parameters of Rabbit Individual Body Weight in the Preweaning Period

**DOI:** 10.3390/vetsci11010014

**Published:** 2023-12-27

**Authors:** Rafik Belabbas, Rym Ezzeroug, Maria De la Luz García, Naouel Feknous, Djamel Talaziza, Maria José Argente

**Affiliations:** 1 Laboratory of Biotechnologies Related to Animal Reproduction, Institute of Veterinary Sciences, University Blida, B.P 270, Road of Soumaa, Blida 9000, Algeria; arymvet@hotmail.com (R.E.); naouelfeknous@yahoo.fr (N.F.); 2 Laboratory of Reaserch “Health and Animal Productions”, Higher National Veterinary School, Road Issad Abes, Oued Smar, Algiers 16200, Algeria; 3 Centro de Investigación e Innovación Agroalimentaria y Agroambiental (CIAGRO-UMH), Universidad Miguel Hernández de Elche, Ctra. Beniel Km 3.2, 03312 Alicante, Spain; mariluz.garcia@umh.es (M.D.l.L.G.); mj.argente@umh.es (M.J.A.); 4 Technical Institute of Animal Breeding, Bab Ali, Alger 16111, Algeria; talaziza1982@gmail.com

**Keywords:** body weight, heritability, kits, rabbit, preweaning

## Abstract

**Simple Summary:**

In order to reduce mortality rates during the suckling period, increasing the kit’s weight during the first days of life could be a selection criterion in rabbit meat breeding programs. The response to selection is directly related to the heritability of the selected trait. The estimates of heritability (*h*^2^) for individual weight were low during the first days of life (0.11 at birth, 0.16 at 5 days, and 0.17 at 7 days) and moderate in the preweaning period (0.21, 0.21, 0.24, and 0.21 at 14, 21, 28, and 35 days, respectively). The weight of the kit at birth showed a strong and positive genetic correlation with weights at 5 and 7 days of age (higher than +0.70). However, the correlation was comparatively low in relation to the remaining weight measurements (less than +0.54). Notably, genetic correlations of weight at 5 and 7 days with the rest of the weight measurements were higher than +0.83. In conclusion, selection for body weight at 5 or 7 days of age would have a significant impact on body weight at birth and at weaning, consequently reducing preweaning losses.

**Abstract:**

The preweaning weight of kits has been related to their mortality during the suckling period. Selecting rabbit kits for individual body weight in the first days of life could be interesting; however, better knowledge of body weight’s heritability during the preweaning period is necessary to determine the opportune moment for selection. A total of 1696 growth records of kits from 81 females of the ITLEV2006 synthetic line were analysed in order to estimate the genetic and non-genetic parameters for individual body weight at birth as well as at 5, 7, 14, 21, 28, and 35 days of age. The estimates of heritability (*h*^2^) for individual weight were between low (0.11 at birth, 0.16 at 5 days, and 0.17 at 7 days) and moderate (0.21, 0.21, 0.24, and 0.21 at 14, 21, 28, and 35 days, respectively). Weight at birth showed a strong and positive genetic correlation with weight at 5 days (+0.79) and 7 days of age (+0.78), but the correlation was low for the rest of the weight measurements (+0.41, +0.49, +0.54, and +0.54 with weight at 14, 21, 28, and 35 days, respectively). Weight at 5 days and 7 days displayed strong and positive genetic correlations with the rest of the weight measurements (higher than +0.83). The values of the common litter effect (*c*^2^) were high, and they increased with age from 0.43 at birth to 0.66 at 35 days of age. The values of the maternal permanent effect (*p*^2^) were low compared to those of the common litter effect (*c*^2^), varying between 0.04 and 0.11. In conclusion, opting to select for body weight at 5 or 7 days of age would yield a greater response compared to selecting for birth weight. This approach would indirectly increase the kits’ weight at birth and at weaning, thereby reducing preweaning losses.

## 1. Introduction

Litter size at birth has been considered the most important trait for evaluating does’ productivity, and significant genetic improvement has been obtained for this trait in the majority of commercial rabbit lines [1]. Nevertheless, the peri- and postnatal mortality rates are rather high, resulting in a total number of alive kits at weaning produced by a doe per year that falls below expectations [2,3]. Several studies have reported that the birth weight of kits is directly linked not only to the rabbits’ growth rate but also to peri- and postnatal survival on the phenotypic scale [4,5,6]. Therefore, to guarantee good viability and growth, it is essential to have newborn kits with a birth weight that is adequately proportionate to that of their littermates. Recently, the practice of selecting rabbits for variability in birth weight within the litter has been reported as an effective method to improve survival [7].

The weight of a rabbit kit is influenced by various factors, including its genotype, maternal effects (such as the age and body weight of the doe, parity order, reproductive rhythm, nutritional status, and uterine environment), and environmental effects (such as ambient temperature, food quality, and breeding management) (see the review by Szendrö et al. [8]). It should be noted that the effect of litter is important, and it should be included in the genetic evaluation of growth parameters. This ensures the avoidance of bias, thus preventing an overestimation of the genetic values of the animals [9]. Genetic evaluation, selection, and the subsequent design of effective breeding programmes are essential for the genetic improvement of any trait of economic interest. Reliable genetic predictions within a population require precise measurement of the heritability of the parameter under study and its genetic correlation with other parameters [1].

In rabbits, extensive research has focused on studying the heritability of body weight at weaning and slaughter [10,11,12]. In general, the heritability of the weight of young rabbits shows low values, typically less than 0.15. This value is similar to the permanent effect of the dam, defined as the ratio between the variance of the female and the total variance. On the other hand, the litter effect, defined as the ratio between the litter variance and total variance, has a higher value compared to the heritability (0.30), indicating a large proportion of the total variance of the trait [1]. However, few studies have analysed the heritability of rabbit weight from birth to weaning. The objective of this study was to estimate the heritability as well as the genetic and phenotypic correlations for the individual weight of kits at birth, 5 days, 7 days, 14 days, 21 days, 28 days, and 35 days, along with evaluating the common litter and permanent effects.

## 2. Materials and Methods

### 2.1. Animals and Management

The present study was conducted at the rabbitry farm of the Technical Institute of Animal Breeding in Algiers, Algeria. All experimental procedures involving animals were approved by the Scientific Council of Biotechnology Laboratory of Animal Reproduction, University Blida1, Institute of Veterinary Sciences, Algeria.

The rabbits used in this study belong to the ITELV2006 synthetic line, the characteristics and breeding programs of which have been previously described by Ezzeroug et al. [12].

Throughout the entire experimental period, the female rabbits were housed in buildings equipped with individual, wired, flat-deck cages (30 cm height × 40 cm width × 70 cm length). The buildings were ventilated with a cooling system, and rabbits were maintained under a consistent light/dark cycle (16L:8D). Rabbits were fed a standard commercial pelleted ad libitum diet (16% crude protein and 15% crude fibre), and water was also available ad libitum from nipple drinkers.

The experimental study was conducted from June to November 2017. The temperature and relative humidity for each month are shown in Table 1. Summer runs from 1 June to 31 August, while autumn spans from 1 September to 30 November.

### 2.2. Experimental Design

The females (n = 82) were initially mated at 20 weeks of age and then again at 10–12 days after parturition. Natural mating was performed using adult males (7–10 months of age) from the same line, with a rhythm of 2 matings per week. Natural mating was employed to trace the genealogy of the kits, in contrast to artificial insemination, which is usually performed with a pool of males’ semen. If females refused mating, they were presented to the male again 7 days later. This implied that some of the females experienced an overlap between gestation and lactation. Pregnancy diagnosis was carried out through abdominal palpation 12 days after mating.

Four days before parturition, cleaned and disinfected nest boxes containing wood shavings were placed into the females’ cages.

One day after parturition, early in the morning (between 7:30 and 8 am), all of the nest boxes were inspected, and their quality was assessed using the method described by Blumetto et al. [13]. This evaluation involved the assessment of extent to which the female used her hair for nest building (categorized as bad if there was no hair in the nest because the female did not prepare it for delivery; intermediate if >50% of the nest had material covered with hair; and excellent if only hair was observed). The location of kindling (inside the nest or in the cage) and the occurrence of cannibalism (presence or absence) were recorded. Cannibalism was considered to occur when at least one kit was devoured by its mother.

The kits were reared by their dams until weaning (35 days of age). Adoptions were not performed.

### 2.3. Recorded Traits

At birth, the litter size was recorded, and the kits were individually identified, sexed, and weighed. Afterwards, the litter size, individual body weights of live kits, and survival rates were recorded at 5, 7, 14, 21, 28, and 35 days for the three first parities. The survival rate was calculated as the number of alive kits at different dates on the total litter size at birth (total number of newborns).

### 2.4. Statistical and Genetic Analyses

A total of 1696 growth records from 208 litters were analysed. The traits studied included individual weight at birth, 5 days, 7 days, 21 days, 28 days, and 35 days of age. The pedigree file included 1815 individuals. Multiple-trait Bayesian analyses were carried out using a linear Gaussian model for growth traits. The following mixed linear model was used:

Y_ijklmnopq_ = μ + P_i_ + L_j_ + S_k_ + N_l_ + C_m_ + B_n_ + G_o_ + m_p_ + c_ijklmop_ + a_ijklmnopq_ + e_ijklmnopq_

where y is the growth trait of animal q, μ is the general mean, P_i_ is the fixed effect of the parity order in which the animal was born (with three levels: 1st, 2nd, and 3rd parity), L_j_ is the fixed effect of lactation status (with two levels: lactating and non-lactating doe at mating), S_k_ is the fixed season of kindling (with two levels: summer and autumn), N_l_ is the fixed effect of nest quality (with three levels: bad, intermediate, and excellent), C_m_ is the fixed effect of cannibalism in kits (with two levels: yes or not), B_n_ is the fixed effect of born kits inside of the nest (with two levels: yes or not), G_o_ is the fixed effect of the gender of kits (with two levels: male and female), m_p_ is the environmental maternal random effect of the overall parity (animal p is the dam of the individual q: 81 levels), c_ijklmop_ is the random effect of the common litter in which the animal q was born (208 levels), a_ijklmopq_ is the random additive genetic value of the animal q, and e_ijklmopq_ is the residual effect.

Heritability (*h*^2^) was defined as the ratio between the additive effect variance and the phenotypic variance, calculated as the sum of variances of the random effects and the error. The common litter effect (*c*^2^) was defined as the ratio between the common litter effect variance and the phenotypic variance. The maternal effect (*p*^2^) was defined as the ratio between the permanent environmental effect variance and the phenotypic variance.

The distribution for additive genetic effects was N (0, A ⊗ G_0_), where A was the additive genetic relationship matrix and G_0_ was the genetic (co)variance matrix between the traits. The distribution for environmental maternal random effects was N (0, I ⊗ M_0_), where I was the identity matrix and M_0_ was the (co)variance matrix of the environmental maternal permanent effects. The distribution for common litter effects was N (0, I ⊗ C_0_), where C_0_ was the (co)variance matrix of the common litter effects, and the residual distribution was N (0, I ⊗ R_0_), where R_0_ was the (co)variance matrix of residuals.

Statistical inferences were derived from samples of the marginal posterior distributions of parameters and variance components obtained through Gibbs sampling, as implemented in the TM programme [14]. The prior distribution for the genetic, environmental maternal, and common litter effects and residual (co)variance matrices is assumed to be an inverted Wishart distribution. Flat priors were used for fixed effects and variance components. The Gibbs sampler was run 1,000,000 rounds, and the first 500,000 rounds were discarded as a warming-up period [14]. A thinning interval of 100 rounds was used to retain sampled values that reduced lag correlation among thinned samples.

## 3. Results

### 3.1. Descriptive Analysis

Table 2 shows simple statistics for litter size traits and individual weights of kits from birth to weaning. The litter size at birth was 8.15, and the number alive kits decreased from 7.37 at birth to 6.05 kits at weaning. At birth, the individual body weight of kits was 54.02 g, which progressively increased to 603.37 g at weaning. The survival rate of kits at birth was 88%. Thereafter, it slowly decreased during the preweaning period to reach a value of 74% at weaning. The individual body weight of kits exhibited higher variability compared to what was observed for the number of alive kits.

### 3.2. Genetic Parameters

The estimated values of heritability (*h*^2^) for individual body weight ranged from 0.11 at birth to 0.24 at 28 days of age (Table 3). The body weight of kits at birth showed a strong and positive genetic correlation with the body weight at 5 days (+0.79) and 7 days of age (+0.78). However, it showed weak but positive correlations with the other measurements (+0.41 with body weight at 14 days, +0.49 with body weight at 21 days, and +0.54 with body weight at 28 and 35 days of age). The genetic correlations between individual weight at 5 days, 7 days, 14 days, 21 days, 28 days, and 35 days of age were positive and high, with values higher than +0.83.

The phenotypic correlations were positive and high among individual weights measured at different time points, ranging from +0.59 to +0.83. However, individual weights measured at 35 days of age showed weak to moderate, yet still positive, correlations with the remaining individual weight measurements.

Table 4 shows the estimated values of the environmental maternal permanent effect (*p*^2^) and their correlations with the individual body weight from birth to weaning. The recorded estimates were low compared to those for the common litter effect, ranging between 0.04 and 0.11. However, the correlations measured were positive and strong for all of the variables, ranging from +0.80 to +0.98.

The estimated values of common litter effects (*c*^2^) for individual weight at birth, 5 days, 7 days, 14 days, 21 days, 28 days, and at weaning are presented in Table 5. The estimates were consistently high regardless of the moment of measurement, ranging from 0.43 for the individual body weight at birth to 0.66 for the individual body weight at weaning. There was no decrease in the common litter effect over time. The correlations were high and positive regardless of the moment of the body weight measurement.

## 4. Discussion

The perinatal weight of kits has been directly linked to preweaning survival; kits with lower birth weights have a lower probability of survival [6,15] due to a reduction in energy reserve and thermoregulatory capacity [16]. In our study, 26% of born kits perished before weaning, which is consistent with the values reported in maternal rabbit lines [17,18]. In agreement with Partridge et al. [19] and Kadi et al. [20], we found that the majority of preweaning losses occurred in the first week of life (73%). Reduced kit survivability not only raises animal welfare concerns but also increases economic costs for rabbit meat production [21]. For these reasons, incorporating strategies to increase the weight of kits in the first days of life could be considered in rabbit breeding programs. However, the success of selection is directly related to its genetic determination [4,22]. In the last decade, new strategies have been proposed to improve the survival of the offspring during the lactation period, i.e., selection for within-litter birth weight variability [7,23] and selection for litter size variability [6].

Numerous studies have explored the genetic determination of the weight of kits at birth and at weaning, yet there are a limited number of studies on the genetic determination of the kits’ weight throughout this entire period. The present study comprises a comprehensive assessment of genetic and environmental influences on kits’ growth and identifies the timing when genetic and/or environmental factors are most influential in order to choose the opportune moment for selection. The heritability (*h*^2^) of individual body weight of kits at birth was low (0.11), which is in agreement with the value reported in rabbits by Testik et al. [24]. However, Varewyck et al. [25] and Argente et al. [4] found higher values of heritability values (0.26 and 0.18, respectively). At weaning, the estimated heritability in this study was similar to that reported by Argente et al. [4]. However, several authors have reported higher values of heritability of body weight at weaning in different rabbit lines (0.42, Sakthivel et al. [26]; 0.34, Shrestha et al. [27]). Additionally, we observed a slight increase in the estimated values of heritabilities from birth to weaning. The heritability estimates for individual body weight at 5 days and 7 days were higher than at birth, suggesting a higher selection response than at birth.

The economic cost of measuring the selected traits is a crucial factor to consider in rabbit selection programs. It could be argued that controlling for litter weight rather than individual weight could reduce this cost. However, estimates of litter weight heritabilities at birth, 21 days, and weaning are less than 0.07 or not significantly different from zero [28,29]. Concerning the genetic correlations, high positive correlations were noted between birth weight and weights recorded at 5 days and 7 days of age (higher than +0.86), whereas for subsequent dates, they were weak to moderate (between +0.41 and +0.54). Our findings are in agreement with the results obtained by Ayayi et al. [30]. These authors have reported positive genetic correlations, with values decreasing from birth to weaning. Our results indicate that selecting for individual body weight at an early age, i.e., such as at birth or during the preweaning period, will lead to weight gain in later ages. This is in agreement with Khalil et al. [31] and Aboukhadiga et al. [32], who reported that selection for high litter weight at birth is generally associated with genetic improvement of this trait at later ages. In addition, genetic correlations between weights at 5 and 7 days and weights at later ages were strong and positive. The high estimated values for genetic correlation could suggest that most of the genes influencing weight at earlier ages may also have an impact on the corresponding trait at later ages [30].

Phenotypic correlations were strong and positive between weights measured at different ages, ranging from +0.59 to +0.83. However, weight at 35 days of age showed weak to moderate correlations with the remaining weights. This may be related to the negative environmental effect on the association of these parameters at this age. Khalil et al. [31] mentioned that a positive phenotypic correlation between two weights does not necessarily indicate that selection for one will result in the improvement of the other. This is because an environmental effect on both weights could be so strong and positively correlated that a negative genetic relationship could be masked. In our study, selection for individual body weight at 5 or 7 days showed high genetic and phenotypic correlations, thereby indicating that its selection will lead to improved individual weight in preweaning, as previously suggested by Odubot and Saumad [33].

The estimates of the common litter effect (*c*^2^) measured in this experiment were high and increased with the age of kits (from 0.43 to 0.66). Moreover, the correlations were strong and positive. Data from the literature concerning the study of individual rabbit body weight and the common litter effect during the preweaning period are scarce. Argente et al. [4] also reported an increase in the estimated values between birth and weaning, ranging from 0.39 to 0.58 in kits born from intact does and from 0.33 to 0.53 in kits born from unilaterally ovariectomized does. The high estimates obtained can be explained by the fact that for each new parturition, the common litter effect is strongly related to the environment, including, in particular, to the female herself, as individuals from the same litter are suckled by the same mother and reared in the same cage [31]. Moreover, the high estimates at 28 days and 35 days compared with those observed at 5 days and 7 days further support our proposal for selecting at early, preweaning ages. Among the non-genetic effects of the female that can influence individual weight, there are the doe’s suckling behavior and milk production aptitude. This effect is linked to the female and remains permanently expressed throughout all of her litters [34]. In addition, the average birth weight decreases with increasing litter size [35,36]. Furthermore, the relationship of litter size with milk production as well as the effect of individual birth weight on both milk production and individual consumption were reported [37]. In particular, milk production increased with litter size, reaching its maximum capacity in response to a litter of seven kits [38]. Moreover, a close connection between the birth weight and the ratio of non-suckled kits was reported by several authors [4,39]. According to Argente et al. [4], this ratio was higher than 50% when the birth weight was below 40 g and lower than 10% in kits with a birth weight higher than 65 g. Our results also showed a decrease in the correlations of the common litter effect between birth weight and weights recorded at the end of weaning. This can be explained by the number of days between weightings. Indeed, the closer the measurement dates, the greater the correlations will be, as the environmental effect is the same.

Estimates of the environmental maternal permanent effect (*p*^2^) are much smaller than those of the common litter effect (*c*^2^). The highest values were noted at 14 days and 21 days (0.11), and the lowest was noted at weaning (0.04). However, all correlations are strong and positive (+0.80 to +0.98). Moreover, the strong correlations indicate the stability of the environmental maternal permanent effect on litter weight throughout the entire lactation period [32].

## 5. Conclusions

The estimated heritability for individual body weight at 5 days and 7 days of age showed higher values than at birth. These traits also showed higher genetic correlations with individual body weight, both at birth and at weaning. Selection based on the weight of kits at 5 or 7 days of age would have a greater response than that based on birth weight. Additionally, the response correlated with the body weight of kits at birth and at weaning would also be greater. Hence, genetic programs could include the kits’ weight at 5 or 7 days of age as a selection criterion in order to improve the survival rates of the kits during the preweaning period.

## Figures and Tables

**Table 1:** Temperature and relative humidity by month.

		Temperature Outside (°C)		Temperature Inside (°C)		Relative Humidity Inside (%)	
		Minimum	Maximum	Minimum	Maximum	Minimum	Maximum
Summer	June	25	30	22	28	21	82
	July	27	39	26	33	21	80
	August	30	35	28	36	23	80
	Average	27.3	34.7	25.3	32.3	21.7	80.7
Autumn	September	24	30	22	27	19	83
	October	21	27	20	23	21	79
	November	16	21	19	23	20	68
	Average	20.3	26.0	20.3	24.3	20.0	76.7

**Table 2:** Number of observations (N), means, and standard deviations (SD) of total litter size, litter size, survival, and individual body weight.

	Traits	N	Mean	SD	Minimum	Maximum
At birth	Total litter size, number of newborns	208	8.15	2.99	1.00	17.00
	Litter size, born-alive kits	208	7.37	3.16	0	17.00
	Litter survival, %	208	88	23	0	100
	Individual body weight, g	1696	54.02	13.14	12.73	96.16
Day 5	Litter size, alive kits at 5 days	208	6.87	3.03	0	15.00
	Litter survival, %	208	83	24	0	100
	Individual body weight of alive kits, g	1431	86.06	23.19	40.31	154.93
Day 7	Litter size, alive kits at 7 days	208	6.63	2.99	0	14.00
	Litter survival, %	208	80	26	0	100
	Individual body weight of alive kits, g	1381	109.18	30.89	52.18	202.38
Day 14	Litter size, alive kits at 14 days	208	6.30	2.91	0	13.00
	Litter survival, %	208	77	27	0	100
	Individual body weight of alive kits, g	1312	192.15	62.23	86.47	417.80
Day 21	Litter size, alive kits at 21 days	208	6.17	2.92	0	13.00
	Litter survival, %	208	75	28	0	100
	Individual body weight of alive kits, g	1284	278.69	97.59	115.11	626.00
Day 28	Litter size, alive kits at 28 days	208	6.08	2.94	0	13.00
	Litter survival, %	208	74	29	0	100
	Individual body weight of alive kits, g	1284	408.86	139.98	179.90	1032.00
Day 35	Litter size, alive kits at 35 days	208	6.05	2.93	0	13.00
	Litter survival, %	208	0.74	0.29	0	100
	Individual body weight of alive kits, g	1266	603.37	203.21	244.52	1161.77

**Table 3:** Heritabilities (diagonal), genetic correlations (above the diagonal), and phenotypic correlations (below the diagonal) for the individual body weight (IBW) from birth to weaning.

	IBW0	IBW5	IBW7	IBW14	IBW21	IBW28	IBW35
IBW0	0.11 (0.09)	+0.79 (0.24)	+0.78 (0.26)	+0.41 (0.45)	+0.49 (0.43)	+0.54 (0.45)	+0.54 (0.49)
IBW5	+0.76 (0.06)	0.16 (0.10)	+0.99 (0.01)	+0.87 (0.16)	+0.83 (0.16)	+0.91 (0.14)	+0.92 (0.11)
IBW7	+0.76 (0.10)	+0.83 (0.06)	0.17 (0.10)	+0.94 (0.09)	+0.91 (0.11)	+0.93 (0.10)	+0.95 (0.10)
IBW14	+0.70 (0.15)	+0.75 (0.14)	+0.79 (0.14)	0.21 (0.10)	+0.97 (0.03)	+0.96 (0.06)	+0.95 (0.08)
IBW21	+0.70 (0.18)	+0.74 (0.14)	+0.77 (0.14)	+0.77 (0.12)	0.21 (0.10)	+0.98 (0.02)	+0.97 (0.04)
IBW28	+0.65 (0.23)	+0.63 (0.22)	+0.63 (0.22)	+0.59 (0.29)	+0.59 (0.37)	0.24 (0.07)	+0.98 (0.02)
IBW35	+0.47 (0.24)	+0.38 (0.25)	+0.46 (0.27)	+0.34 (0.45)	+0.27 (0.47)	+0.54 (0.32)	0.21 (0.09)

IBW0: Individual birth weight at birth. IBW5: Individual birth weight at 5 days. IBW7: Individual birth weight at 7 days. IBW14: Individual birth weight at 14 days. IBW21: Individual birth weight at 21 days. IBW28: Individual birth weight at 28 days. IBW35: Individual birth weight at 35 days (weaning). Standard errors between parentheses.

**Table 4:** Estimation of environmental maternal permanent effects (*p*^2^) (diagonal) and their correlations for the individual body weight (IBW) from birth to weaning.

	IBW0	IBW5	IBW7	IBW14	IBW21	IBW28	IBW35
IBW0	0.09 (0.05)	+0.93 (0.12)	+0.86 (0.20)	+0.85 (0.28)	+0.81 (0.19)	+0.80 (0.15)	+0.80 (0.15)
IBW5		0.06 (0.04)	+0.98 (0.04)	+0.93 (0.14)	+0.91 (0.17)	+0.91 (0.16)	+0.87 (0.28)
IBW7			0.09 (0.06)	+0.97 (0.05)	+0.95 (0.09)	+0.95 (0.09)	+0.92 (0.17)
IBW14				0.11 (0.06)	+0.98 (0.03)	+0.97 (0.05)	+0.92 (0.16)
IBW21					0.11 (0.06)	+0.98 (0.03)	+0.95 (0.10)
IBW28						0.06 (0.04)	+0.97 (0.06)
IBW35							0.04 (0.03)

IBW0: Individual birth weight at birth. IBW5: Individual birth weight at 5 days. IBW7: Individual birth weight at 7 days. IBW14: Individual birth weight at 14 days. IBW21: Individual birth weight at 21 days. IBW28: Individual birth weight at 28 days. IBW35: Individual birth weight at 35 days (weaning). Standard errors between parentheses.

**Table 5:** Estimation of common litter effect (*c^2^*) (diagonal) and its correlations for the individual body weight (IBW) from birth to weaning.

	IBW0	IBW5	IBW7	IBW14	IBW21	IBW28	IBW35
IBW0	0.43 (0.05)	+0.83 (0.03)	+0.79 (0.04)	+0.72 (0.05)	+0.67 (0.06)	+0.63 (0.07)	+0.54 (0.07)
IBW5		0.46 (0.05)	+0.95 (0.01)	+0.84 (0.03)	+0.72 (0.15)	+0.75 (0.04	+0.63 (0.06)
IBW7			0.47 (0.07)	+0.91 (0.02)	+0.83 (0.03)	+0.78 (0.04)	+0.68 (0.05)
IBW14				0.55 (0.06)	+0.96 (0.01)	+0.88 (0.02)	+0.77 (0.04)
IBW21					0.55 (0.06)	+0.95 (0.01)	+0.84 (0.03)
IBW28						0.63 (0.05)	+0.92 (0.01)
IBW35							0.66 (0.05)

IBW0: Individual birth weight at birth. IBW5: Individual birth weight at 5 days. IBW7: Individual birth weight at 7 days. IBW14: Individual birth weight at 14 days. IBW21: Individual birth weight at 21 days. IBW28: Individual birth weight at 28 days. IBW35: Individual birth weight at 35 days (weaning). Standard errors between parentheses.

## Data Availability

The data generated and analyzed during this study are included in this article.
